# Analysis of sex-specific disease patterns associated with human lifespan

**DOI:** 10.1007/s11357-024-01470-z

**Published:** 2025-01-15

**Authors:** Sara Cruces-Salguero, Joaquim Sol, Igor Larrañaga, Reinald Pamplona, Javier Mar, Mariona Jove, Ander Matheu

**Affiliations:** 1 Cellular Oncology Group, Biogipuzkoa (Biodonostia) Health Research Institute, San Sebastian, Spain; 2 Catalan Health Institute, Lleida Research Support Unit, Fundació Institut Universitari Per a La Recerca en Atenció Primària de Salut Jordi Gol I Gurina, Lleida, Spain; 3 Department of Experimental Medicine, University of Lleida-Lleida Biomedical Research Institute, Lleida, Spain; 4 Osakidetza Basque Health Service, Debagoiena Integrated Healthcare Organisation, Research Unit, Mondragon, Spain; 5 Kronikgune Institute for Health Services Research, Barakaldo, Spain; 6 Epidemiology and Public Health Department, Biodonostia Health Research Institute, San Sebastián, Spain; 7 IKERBASQUE, Basque Foundation for Science, Bilbao, Spain; 8 Centro de Investigación Biomédica en Red de Fragilidad y Envejecimiento (CIBERfes), Carlos III Institute, Madrid, Spain

**Keywords:** Health span, Sex-specific disease patterns, Lifespans

## Abstract

Disability and multimorbidity increase with aging and constitute a challenge for the health system. However, different individuals display different aging trajectories, and understanding the underlying relationships between lifespan and disease is fundamental for identifying the different patterns in human lifespan. A previous study explored associations between lifespan and age of onset of diseases of different organ systems, prevalence of escapers, and percentage of life free of disease (health span), comparing them between genders in Catalonian population. In this retrospective study, we analyzed a cohort of 41,063 old individuals (20,722 men, with a mean lifespan of 79 years [50–109], and 20,341 women, with a mean lifespan of 84 years [50–109]) deceased between 2014 and 2019 in Gipuzkoa (Basque Country, Spain) taken from the Basque Health Service electronic health records data lake and analyzed and contrasted the above cited characteristics. Longevity, defined by age above the mean lifespan of the population, showed a protective effect against developing diseases, with higher lifespans delaying the onset of all diseases both at single and multisystemic levels. Furthermore, individuals with the lowest and highest lifespans were the ones presenting the healthier profiles, with fewer systems altered. There were specific differences according to sex, with women exhibiting decreased mortality risk despite having greater multimorbidity and men having less multimorbidity. In addition, men reached the highest lifespans with a smaller number of comorbidities. These results define the aging trajectories of the Basque population, extend the characterization of the human aging, and provide insight into sex-specific differences in multimorbidity and survival risk.

**Supplementary Information:**

The online version contains supplementary material available at 10.1007/s11357-024-01470-z.

## Introduction

Life expectancy has increased over the last years, and the trend is expected to continue on the rise [1, 2]. However, the rise in lifespan is not necessarily accompanied by an increase in health life expectancy, or health span. The repercussions in health and disease related to the increase in life expectancy have been primarily addressed by two theories. The “expansion of morbidity” theory implies that the extra years of life do not come with extra years of health span, therefore increasing the occurrence of diseases and disability [3]. In fact, aging has been reported as the main cause of chronic diseases [4, 5], which in turn constitute more than 80% of mortality [6, 7] and disability [6]. On the other hand, it would be expected that this increase in life expectancy due to health-related advances would also imply an increase on health span; this could be related to the “compression of morbidity” theory [6]. In this direction, studies in centenarian populations showed that a large proportion of centenarians delay significantly their disease experiences or even completely escape from these morbidities, showing that morbidity and disability can be compressed toward the end of life [8, 9]. These results postulated centenarians as a model of healthy aging with extended lifespan and health span [10]. However, studies also showed that there is heterogeneity between the group of centenarians with significant differences between individuals [11], and cases from both theories existing in different populations [12]. In addition to these theories, it has also been suggested that each individual could present different trajectories of aging, with diseases and disability starting at different ages in function of individual vulnerability and resilience [13]. In this direction, Sol et al. recently published a study assessing the relationship between human lifespan and disease using clinical data from a large number of deceased individuals in Catalonia (Spain), finding associations between the time of onset of disease, health span, and individual lifespan, discerning specific disease patterns for systems and gender [14]. Among their main findings, they saw that the age of onset of the first disease is delayed as lifespan increased, that the prevalence of escapers in young and long-lived individuals was higher than in the rest of the older population, and that women presented fewer comorbidities [14].

In 2023, Spain had the eighth highest life expectancy in the world, and the third in Europe [15].

Basque Country is distributed along the western edge of the Pyrenees, spanning across Spanish and French territories. Over the last decades, research placed Basques as an isolated and unique population due to their singular biological, anthropological, and cultural traits. Among the former, the high frequency of the Rh-negative blood group [16], the R1441G mutation in *LRRK2* gene associated with Parkinson’s Disease [17], and additional genetic marks [18, 19] highlight that Basques are particularly differentiated within the Spanish and European genetic context. Among the latter, the existance of Euskera, a non-Indo-European language with no close relationship to any other extant language [20], which enhanced the isolation, and the Basque gastronomy are the most remarkable characteristics.

In relation to longevity and aging, the region of the Basque Country is one of the most aged, with a mean life expectancy of 83 years in 2022 [21] and more than 20% of the population being over 65 years old [22]. In this region, population aging has been associated with multimorbidity whose prevalence increases along with age [23]. However, using the electronic health records of deceased individuals of the last 10 years, we observed that the centenarian population of the Basque Country presented a lower number of aging-associated diseases, comorbidities, and reduced need for clinical resources [24]. Some of these findings agreed with the results of the study of Sol et al. [14]; however, in the Catalan database, they also analyzed and described lifespan- and health span-specific features that had not been previously assessed on the Basque population.

Based on this data, and taking advantage of the similarities between Basque and Catalan databases of both health systems, we decided to replicate the study performed by Sol et al. [14] in the database of the Basque Country health system Osakidetza. In this line, EHR data from Basque Health Service follows a National Health System (NHS) Beveridge type system and has high-quality performance indicators [25], an important aspect in order to complete robust analysis with it. We studied a cohort of 41,063 deceased individuals with a mean lifespan of 82 years, and we assessed the underlying relationships between human lifespan and pathologies. The interpretation of this analysis allowed us to define lifespan and disease relationships in the Basque population, to reinforce part of the results obtained by Sol et al. regarding human aging trajectories in a new cohort, and to highlight the differences between Basque and Catalan old populations in terms of lifespan and disease.

## Materials and methods

### Study population

An observational study was carried out with real-world data, retrospectively analyzing aging-related diseases and their effect on lifespan in the Basque population. This study was based on old deceased individuals in Gipuzkoa between 2014 and 2019 recorded in the data lake of the Basque Health Service, which contains information from 2004 onward when the electronic system was implemented. A data lake is a collection of various data assets that are stored within a Hadoop ecosystem with minimal change to the original format or content of the source data. Prior 2004, the database has records from before (there are diagnosis from 1920, for instance), but the transition to the electronic system might have introduced some bias due to lost records. All the information registered in the database was anonymized and included demographic and clinical data.

First, the quality of the data was evaluated to delete patients whose data did not fulfill the quality criteria. Individuals whose date of birth and/or death did not have a valid format (dd/mm/yyyy) or had missing values were excluded, as well as patients whose date of birth was posterior to the date of death or who had more than one date of death.

After data curation, 41,137 individuals remained. Individuals younger than 50 years (*n* = 74) were excluded (final *n* = 41,063). For each individual, information about sex, age, province, status in nursing home, and diagnoses was included. Age was calculated as the difference between the date of death and the date of birth.

The database included information about the diagnoses determined in all the episodes of primary care, emergency, outpatient, and in-hospital care and was recorded through the International Classification of Diseases, Ninth Revision (ICD-9), and Tenth Revision (ICD-10) diagnosis codes. We selected age-related diseases included in the study performed by Sol et al. [14]: age-related chronic conditions [26] and frailty-related diseases [27] divided into eight groups: neoplasms, endocrine, nutritional and metabolic diseases, diseases of the nervous system, diseases of the circulatory system, diseases of the respiratory system, diseases of the digestive system, diseases of the musculoskeletal system and connective tissue, and diseases of the genitourinary system. This classification was performed through regular expressions. For each category, we saved the date of the first diagnosis. Diagnoses after patients’ date of death were discarded.

### Statistical analysis

We followed the methodology of Sol et al. [14] to analyze our data. Descriptive statistics were applied to analyze the Basque population in terms of lifespan, gender, province, and status in nursing home. Participants older than 100 years (*n* = 197) were included in the 100 years old group, due to the small sample size. In the tables, data was classified by age-decade.

Survival analysis was performed to assess the relationship between age of first diagnosis, lifespan, and sex. We employed Cox regression to predict the onset of diseases in function of lifespan, sex, and their interaction. Lifespan was centered in the mean before performing this analysis. Results were presented as hazard ratios (HR) with their respective 95% confidence intervals. For each of the eight disease categories, an independent regression was performed. We also evaluated the onset of the first disease independently from its category. The results were represented through Kaplan–Meier curves, using truncated lifespan as a categorical variable, for each gender. We repeated the same approach for evaluating comorbidities, taking the age of onset of an *n*th disease (*n* = 2–8). Kaplan–Meier curve for the eighth comorbidity was not presented due to the small sample size.

Next, we assessed the prevalence of escapers, defined as individuals who died without a specific disease, for each category, gender, and lifespan. We represented locally estimated scatterplot smoothing (LOESS) curves for each category and gender across lifespans. We used *k*-means clustering to group similar patterns between disease categories, selecting the optimal number of clusters through the higher average silhouette width method, for a number of clusters from 2 to 7. We also evaluated the number of systems free of disease, representing the cumulative prevalence for each lifespan, LOESS curves for each number of systems and gender, and the mean number of systems affected for each lifespan and gender.

Evaluation of health span was performed in those individuals with disease. We computed the median percentage of life free of disease for each category, gender, and lifespan and represented it through LOESS curves. As in the previous analysis, we used *k*-means clustering to classify diseases into similar groups.

Finally, we evaluated if the previous aspects were able to describe our population in terms of lifespan. For that, we performed a multiple factor analysis (MFA). We created four groups of variables: sex, escaping the disease (for each of the categories), number of systems free of disease, and percentage of life free of disease (for each of the categories). We represented the lifespan of a random sample of 10% of our population explained by the first and second components obtained by the MFA, and we evaluated the importance of each of the previous groups of variables in explaining lifespan.

The building and transformation of the database were performed in Python 3.9. All the statistical analyses were performed in RStudio, v.4.2.2 (URL: https://www.r-project.org/). S*urvival *[28] and *survminer* packages were used for survival analysis, and *factoextra* and *FactoMineR *[29] for MFA.

### Ethics

This study was approved by the Basque Clinical Research Ethics Committee (CEIm-E code PI2020206) and adhered to the tenets of the Declaration of Helsinki by the World Medical Association regarding human experimentation.

## Results

### Demographic data

We studied a population of 41,063 individuals deceased in Gipuzkoa (region of the Basque Country) from the beginning of 2014 to December 31, 2019. The mean age of the population was 82 years, with the youngest individual deceased at 50 years and the oldest at 109 years. The proportion of women and men was almost the same (49.54% women vs 50.46% men). At lower lifespans, the proportion of men was higher (65% vs 35% at 60 s), with this trend reverting until the centenarians, where women far surpassed men (85% vs 15%). The demographic characteristics of the population were summarized in Table 1.

**Table 1:** Demographic description of the population

Death decade	All (*N* = 41,063)	50–59 (*N* = 2431)	60–69 (*N* = 4337)	70–79 (*N* = 7180)	80–89 (*N* = 16,282)	90–99 (*N* = 10,516)	100 + (*N* = 317)
Sex							
Women	20,341 (49.54%)	849 (34.92%)	1,389 (32.03%)	2,515 (35.03%)	8,123 (49.89%)	7,194 (68.41%)	271 (85.49%)
Men	20,722 (50.46%)	1,582 (65.08%)	2,948 (67.97%)	4,665 (64.97%)	8,159 (50.11%)	3,322 (31.59%)	46 (14.51%)
Province							
Gipuzkoa	39,946 (97.28%)	2,350 (96.67%)	4,194 (96.70%)	6,959 (96.92%)	15,842 (97.3%)	10,293 (97.88%)	308 (97.16%)
Rest of Basque Country	1,023 (2.49%)	71 (2.92%)	125 (2.88%)	213 (2.97%)	411 (2.52%)	196 (1.86%)	7 (2.21%)
Others	94 (0.23%)	10 (0.41%)	18 (0.42%)	8 (0.11%)	29 (0.18%)	27 (0.26%)	2 (0.63%)
Residence							
Yes	5,190 (12.64%)	68 (2.8%)	166 (3.83%)	563 (7.84%)	2,286 (14.04%)	2,031 (19.31%)	76 (23.97%)
No	35,873 (87.36%)	2,363 (97.2%)	4,171 (96.17%)	6,617 (92.16%)	13,996 (85.96%)	8,485 (80.69%)	241 (76.03%)

Data is presented grouped by age of death decade

### Age of onset of diseases

**Fig. 1:**
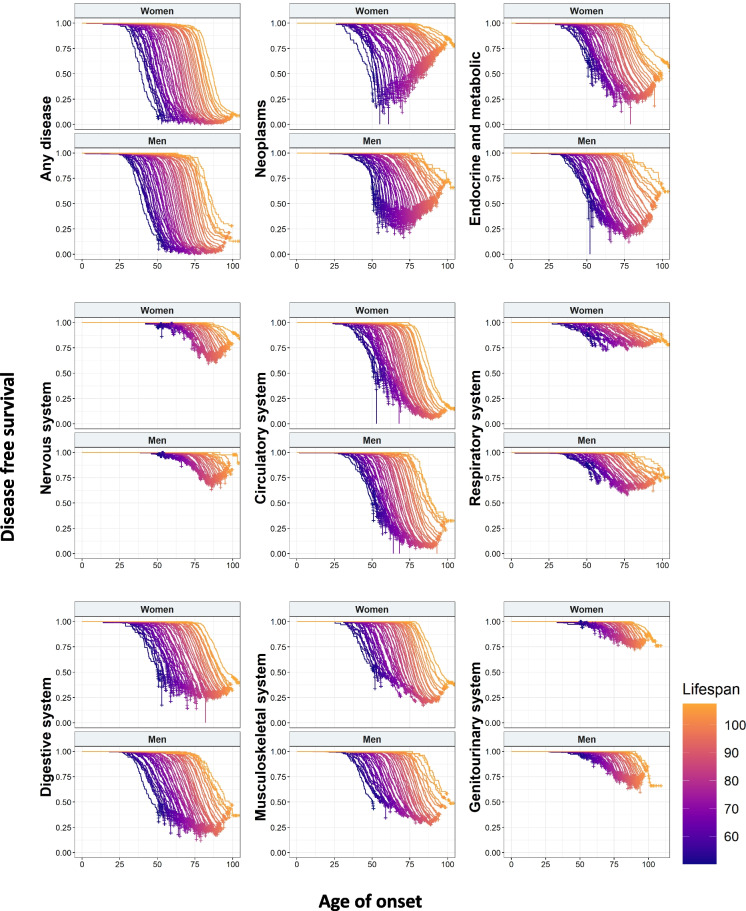
Kaplan–Meier curves of the age of onset of disease for different disease groups in function of lifespan and sex

First, we performed survival analysis in order to study the relationship between the age of first diagnosis and lifespan. For that, we built Kaplan–Meier curves for each ICD-10 disease category and sex. We observed differences between diseases with neoplasms and circulatory, digestive endocrine, and musculoskeletal system having the higher incidence. Noteworthy, we obtained a continuous spectrum, independently of the disease, in which the higher the lifespan, the higher the age of onset (Fig. 1). Some diseases, such as neoplasms and diseases of respiratory and musculoskeletal systems, displayed different patterns between women and men. We also saw that some diseases, such as those related to the circulatory system, had a higher incidence when increasing lifespan, while neoplasms, particularly in the case of women, showed the opposite behavior (Fig. 1).

Next, we analyzed the HR provided by the Cox regression to test the effect of lifespan and sex in the onset of each disease categories. For all the diseases, lifespan had a protective effect: the higher the lifespan, the lower the HR (Table 2). Regarding sex, it also had a significant HR for every category, with women showing a protective effect for every disease except for those belonging to the nervous and musculoskeletal systems. When assessing interactions between lifespan and sex, we found that for most disease groups, women at shorter lifespans had higher HR for developing diseases (Table 2).

**Table 2:** Cox regression for each disease group

Disease group	HR (95% CI) for age of death	HR (95% CI) for sex (women)	HR (95% CI) for age of death: sex (women)
Any disease	0.879 (0.878–0.880)***	0.989 (0.969–1.009)	1.005 (1.003–1.007)***
Neoplasms	0.847 (0.845–0.849)***	0.762 (0.740–0.785)***	0.975 (0.973–0.978)***
Endocrine, nutritional, and metabolic diseases	0.893 (0.891–0.895)***	0.973 (0.950–0.997)*	1.011 (1.008–1.013)***
Diseases of the nervous system	0.836 (0.831–0.841)***	1.284 (1.227–1.344)***	0.996 (0.991–1.002)
Diseases of the circulatory system	0.897 (0.896–0.899)***	0.971 (0.950–0.992)**	1.013 (1.011–1.016)***
Diseases of the respiratory system	0.910 (0.907–0.912)***	0.610 (0.586–0.636)***	0.998 (0.994–1.002)
Diseases of the digestive system	0.889 (0.887–0.891)***	0.898 (0.877–0.920)***	1.005 (1.002–1.007)***
Diseases of the musculoskeletal system and connective tissue	0.895 (0.893–0.896)***	1.356 (1.323–1.390)***	1.009 (1.006–1.011)***
Diseases of the genitourinary system	0.804 (0.799–0.809)***	0.685 (0.652–0.720)***	1.004 (0.999–1.010)

Results are presented as HR with their respective 95% CI

Significant results are displayed in bold

*p < 0.05; **p < 0.01; ***p < 0.001

We repeated the same approach to evaluate the age of onset of individuals with multiple systems affected. We assessed the age of onset of the *n*th disease category affected, from the second to the eighth. As in the previous analysis, the Kaplan–Meier curves showed the same delay on the onset as the lifespan increased, indicating that this effect was not specific for single diseases. Furthermore, as the number of affected systems increased, the incidence decreased (Supplementary Fig. 1). In Cox regression, we saw that once again lifespan had a protective effect, exhibiting lower HR as the number of systems involved increased. On the other hand, women showed protection against having multiple systems affected, a protection which increased along with the number of systems affected (Supplementary Table 1). These results showed that the most long-lived individuals displayed longer health spans being women particularly protected.

### Prevalence of escapers

**Fig. 2:**
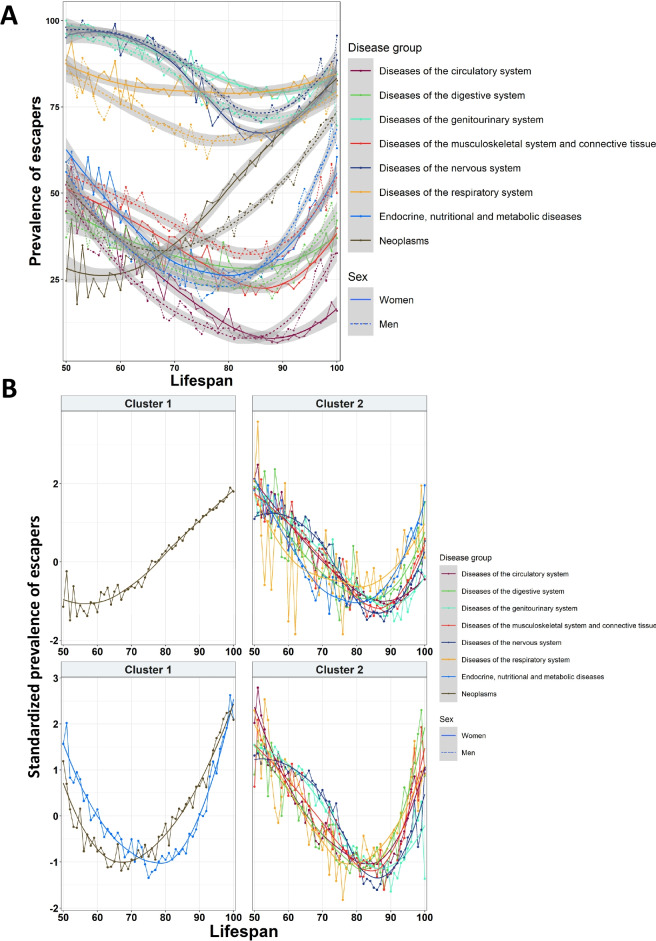
Trajectories of escapers for each disease group in function of lifespan and sex. **A** Overall trajectories of the prevalence of escapers for each disease group. **B** Analysis of cluster for escaper trajectories for different disease groups in women (first row) and men (second row)

Next, we evaluated the capacity to avoid diseases for each lifespan. We defined “escapers” as those individuals who died without a specific disease; for instance, escapers for neoplasms were individuals that died without having a diagnosis of neoplasm, even if they had diseases from other categories. We represented the trajectory of the prevalence of escapers at different lifespans for each disease group and gender. All diseases reached a minimum of escapers at 70–90 years. Furthermore, most categories showed similar prevalences at the lowest and highest lifespans. We observed some extreme patterns, such as that less than 10% of the population with a lifespan of 80–90 years was able to avoid circulatory diseases. On the other hand, diseases of the nervous and genitourinary systems had almost no incidence at lifespans of 50–60 years (Fig. 2A).

There were some differences associated with sex, with women having a higher prevalence of escapers in the majority of diseases. The cluster analysis revealed that, in the case of women, all disease categories followed a similar trend of escapers except for neoplasms. Coherently with the survival analysis, neoplasms showed a lower incidence of escapers at younger lifespans, and the prevalence increased along with the lifespan, which indicated that women who reached higher ages were able to avoid this disease. On the other hand, all the other groups of diseases showed a concave-shaped pattern, reaching the minimum number of escapers at a lifespan of around 85 years. In the case of men, we observed a similar pattern, but in this case, metabolic diseases were included in the same cluster as neoplasms. In men, both diseases reached a minimum prevalence of escapers before a lifespan of 80 years, and at higher lifespans, the prevalence of escapers was higher when compared to the other diseases (Fig. 2B).

**Fig. 3:**
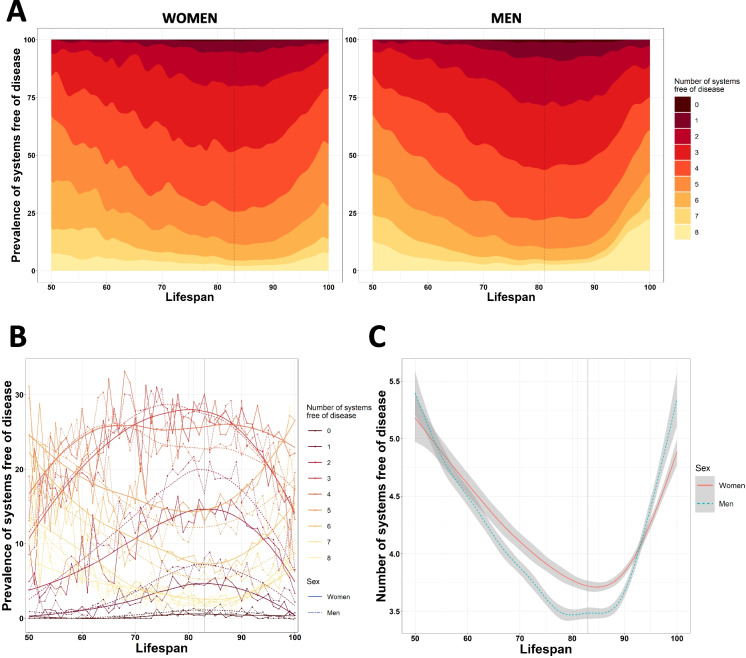
Trajectories of the number of systems free of disease. **A** Cumulative prevalence of individuals with each number of systems free of disease for women and men in function of lifespan. **B** Overall trajectories of the prevalence of individuals with each number of systems free of disease in function of lifespan and sex. **C** Trajectories of the number of systems free of disease for women and men in function of lifespan

When assessing the multisystem involvement, we saw a similar pattern: around 85 years, the number of systems free of disease reached their minimum, while the lowest and highest lifespans exhibited the maximum. More specifically, the minimum number of systems free of disease was reached at a lifespan of 83 years for women and 81 years for men (Fig. 3A–B). We also observed differences in the number of systems free of disease for both sexes across different lifespans, with women maintaining an overall lower number of comorbidities. However, men reached higher lifespans with fewer systems affected (Fig. 3C). The prevalences of the number of systems free of disease for each age decade were summarized in Table 3. These results showed that the long-lived individuals presented more rates of disease escapers with women presenting particularly lower number of comorbidities. Indeed, 10% of centenarians were free of diseases in contrast to only 2–3% individuals between 70 and 89 years-old groups. The comparison with Catalonian data revealed that the mean number of systems free of disease for individuals deceased between 50 and 80 years was slightly lower for both sexes in our cohort.

**Table 3:** Prevalence of escapers according to the number of systems free of disease and age of death decade

Death decade	All		50–59		60–69		70–79		80–89		90–99		100 +	
	Women (*N* = 20,341)	Men (*N* = 20,722)	Women (*N* = 849)	Men (*N* = 1582)	Women (*N* = 1389)	Men (*N* = 2948)	Women (*N* = 2515)	Men (*N* = 4665)	Women (*N* = 8123)	Men (*N* = 8159)	Women (*N* = 7194)	Men (*N* = 3322)	Women (*N* = 271)	Men (*N* = 46)
Systems free of disease (continuous)														
	4 ± 1.59	3.83 ± 1.7	4.92 ± 1.62	4.87 ± 1.67	4.32 ± 1.59	4.15 ± 1.59	3.92 ± 1.6	3.65 ± 1.59	3.74 ± 1.52	3.5 ± 1.61	4.12 ± 1.6	4.07 ± 1.89	4.96 ± 1.6	4.96 ± 1.94
Systems free of disease (categorical)														
0	71 (0.35%)	151 (0.73%)	1 (0.12%)	0 (0%)	0 (0%)	7 (0.24%)	12 (0.48%)	33 (0.71%)	37 (0.46%)	85 (1.04%)	20 (0.28%)	26 (0.78%)	1 (0.37%)	0 (0%)
1	691 (3.4%)	1,055 (5.09%)	6 (0.71%)	15 (0.95%)	23 (1.66%)	55 (1.87%)	89 (3.54%)	260 (5.57%)	383 (4.73%)	567 (6.95%)	187 (2.6%)	157 (4.73%)	2 (0.74%)	1 (2.17%)
2	2,490 (12.24%)	3,257 (15.72%)	38 (4.48%)	83 (5.25%)	117 (8.42%)	331 (11.23%)	327 (13%)	778 (16.68%)	1,183 (14.56%)	1,589 (19.48%)	816 (11.34%)	473 (14.24%)	9 (3.32%)	3 (6.52%)
3	5,018 (24.67%)	5,261 (25.39%)	137 (16.14%)	260 (16.43%)	320 (23.04%)	714 (24.22%)	686 (27.28%)	1,311 (28.1%)	2,188 (26.94%)	2,185 (26.78%)	1,651 (22.95%)	783 (23.57%)	36 (13.28%)	8 (17.39%)
4	5,187 (25.5%)	4,801 (23.17%)	177 (20.85%)	331 (20.92%)	379 (27.29%)	767 (26.02%)	609 (24.21%)	1,105 (23.69%)	2,133 (26.26%)	1,861 (22.81%)	1,829 (25.42%)	728 (21.91%)	60 (22.14%)	9 (19.57%)
5	3,488 (17.15%)	3,008 (14.52%)	176 (20.73%)	363 (22.95%)	242 (17.42%)	502 (17.03%)	383 (15.23%)	603 (12.93%)	1,221 (15.03%)	1,032 (12.65%)	1,394 (19.38%)	500 (15.05%)	72 (26.57%)	8 (17.39%)
6	1,928 (9.48%)	1,570 (7.58%)	157 (18.49%)	242 (15.3%)	162 (11.66%)	319 (10.82%)	235 (9.34%)	327 (7.01%)	591 (7.28%)	450 (5.52%)	735 (10.22%)	228 (6.86%)	48 (17.71%)	4 (8.7%)
7	771 (3.79%)	673 (3.25%)	103 (12.13%)	155 (9.8%)	83 (5.98%)	133 (4.51%)	93 (3.7%)	117 (2.51%)	197 (2.43%)	142 (1.74%)	278 (3.86%)	119 (3.58%)	17 (6.27%)	7 (15.22%)
8	697 (3.43%)	946 (4.57%)	54 (6.36%)	133 (8.41%)	63 (4.54%)	120 (4.07%)	81 (3.22%)	131 (2.81%)	189 (2.33%)	248 (3.04%)	284 (3.95%)	308 (9.27%)	26 (9.59%)	6 (13.04%)

Results are presented as number of escapers and percentages (%) with respect to their age group

### Assessment of health span

The next step was to assess the health span of individuals, or the period of lifespan that individuals had without diseases. For that, we focus on individuals that had at least one disease. We evaluated the percentage of life free of disease, defined as the years from birth until the time of onset of the first disease divided by the lifespan, for each category, lifespan, and sex.

**Fig. 4:**
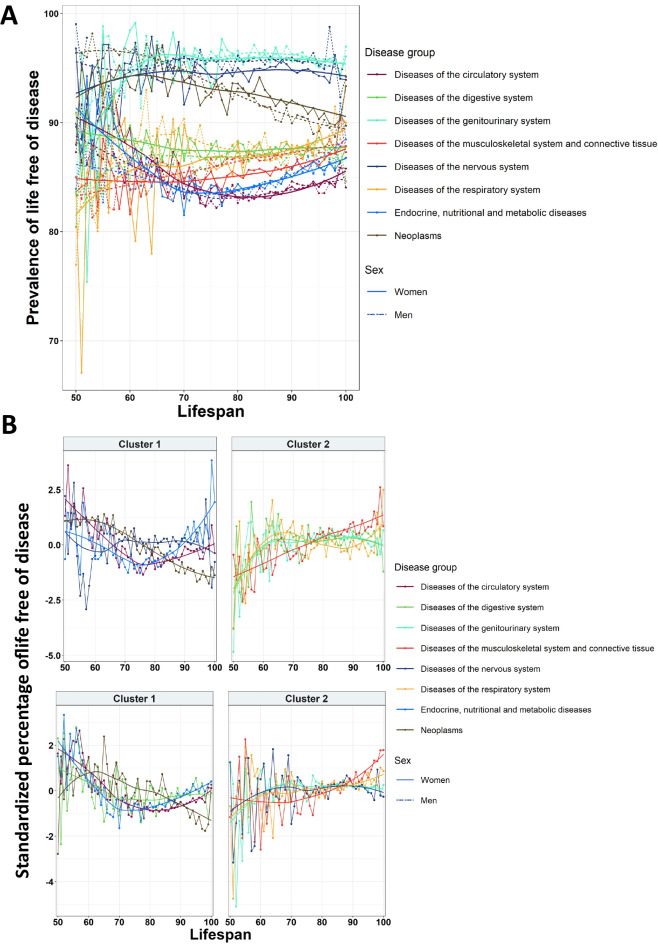
Trajectories of the life free of disease. **A** Overall trajectories of the prevalence of life free of disease for each disease group in function of lifespan and sex. **B** Analysis of cluster of the trajectories of life free of disease for the different disease groups in men (first row) and women (second row)

First, we observed different patterns both among systems and among genders. There was a notable overlap among the different diseases at lower lifespans; however, as lifespan increased, two main trajectories could be distinguished: those that maintained a higher prevalence of life free of disease, with more than 90%, and those with a lower prevalence, which reached around 85%. Diseases from both trajectories seemed to converge at higher lifespans. Notably, at lower lifespans, men exhibited a higher percentage of life free of disease for neoplasms when compared to women, while metabolic diseases displayed the opposite behavior (Fig. 4A).

The cluster analysis revealed two main clusters for women and two clusters for men. In the case of women, in the first cluster, neoplasms, diseases of the circulatory, digestive, and metabolic systems were grouped. Except for neoplasms, diseases of this cluster reached their minimum prevalence of health span at a lifespan of 70 years, while neoplasms showed a decrease in health span as lifespan increased. In the second cluster, there were diseases of the musculoskeletal, respiratory, nervous, and genitourinary systems, which had a tendency of increasing their health span period along with lifespan. In the case of men, diseases of the nervous system were in the first cluster, and diseases of the digestive system were in the second one, while the rest remained the same (Fig. 4B). These results described differences on health span between genders.

### Contributions to lifespan

**Fig. 5:**
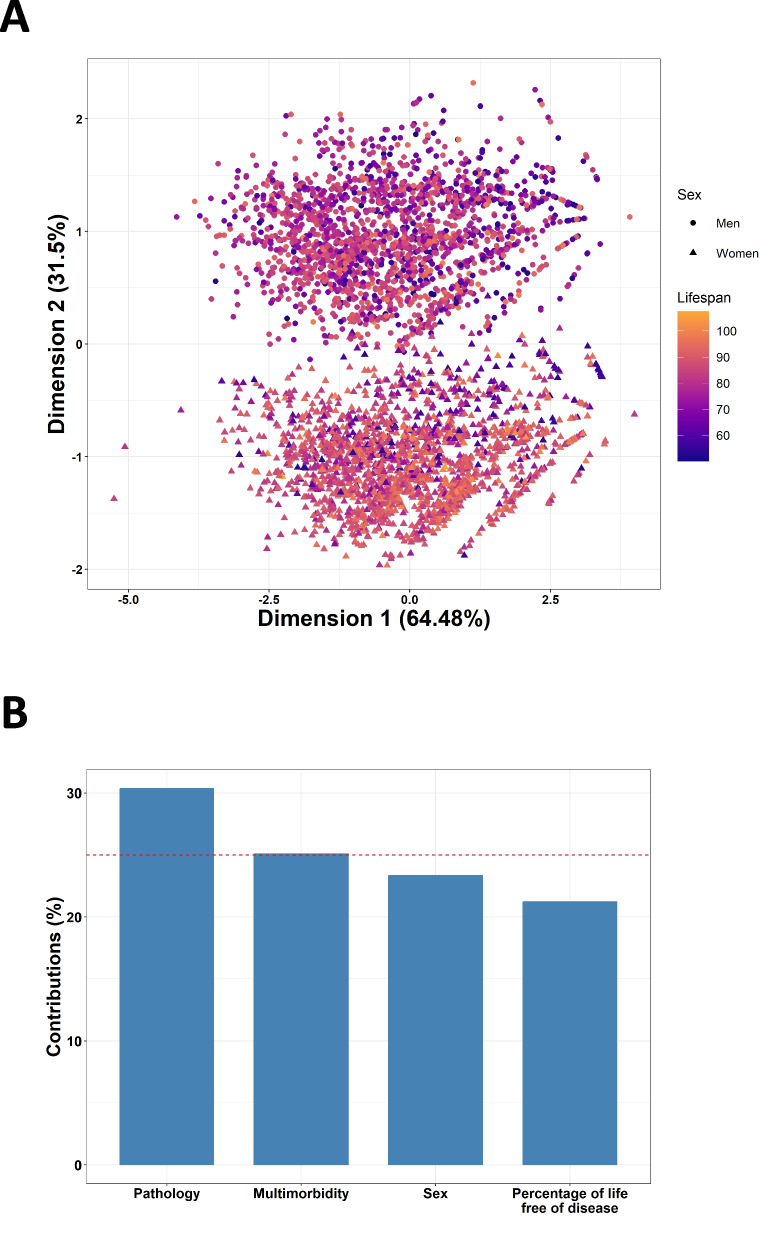
Multiple factor analysis of the assessed variables. **A** Representation of a random sample of 10% of the population in terms of lifespan and sex according to the first and second dimensions of the MFA. **B** Contribution of the groups of variables to the MFA. The red line indicates the expected value considering uniform distribution

Finally, we evaluated the capacity of the previously assessed variables to explain lifespan. For that, we performed an MFA. We included each pathology, sex, the number of systems free of disease, and the percentage of life free of disease for each category as variables. We represented a random sample of the 10% of the population in terms of lifespan using the first two dimensions of the MFA. First, we saw that our variables were able to discern the differences in lifespan, since the distribution of ages of death followed a pattern, with the lifespan decreasing along the vertical axis. We also observed two differentiated clusters of individuals, with the first one being all women and the second one being men (Fig. 5A). When assessing the contribution of the variables to the algorithm, the different pathologies were the group of variables that most contributed to explaining lifespan, followed by multimorbidity, sex, and the percentage of life free of disease (Fig. 5B).

## Discussion

Epidemiological studies involving older individuals in aging research have been historically performed taking advantage of selected cohorts and questionnaires and self-reported data. However these studies have limitations as they might not represent the total population, they can suffer the problem of loss to follow-up, they are usually expensive and they cannot offer the same information of medical records, specially concerning diseases and diagnoses [30]. Additionally, studies have been completed in groups based on ranges of crude age, which is an obvious important aspect on aging research and when considering phenotypic and functional changes in health and disease, but do not take into account intrinsic biological characteristics and environmental factors [31, 32]. Centenarians are an example of this issue with heterogeneity among them and cases from both compression (the “delayers” and “escapers” profiles) and expansion (the “survivors”) of morbidity theories existing in different populations [12]. In this regard, a recent study by Sol et al. have designed and completed a retrospective analysis of lifespan and disease in the oldest people taking into consideration individual trajectories and a pragmatic approach using electronic health records [14]. Moreover, this type of information and approaches have been used to develop disease and risk score predictive models [33, 34].

In this work, we unraveled disease trajectory patterns and their relationship with lifespan and health span in the old population of the Basque Country, following the methodology of Sol et al. and extrapolating some of the results obtained in Catalonia region [14]. In our cohort, we observed that as lifespan increased, the age of onset of all diseases also increased, exhibiting a continuous delay. Furthermore, we saw this delay in the onset of subsequent systems affected, with age showing a stronger protective effect as the number of affected systems increased. Therefore, long-lived individuals were able to postpone the onset of all diseases both at individual and multisystemic levels. Studies focused on centenarians [12] and long-lived individuals [35] already reported this profile of “delayers,” with our data expanding these results to the rest of the older population.

In the evaluation of escapers, we observed that the highest prevalence of escapers was found at the shortest and highest lifespans, for the majority of diseases individually and also at a multisystemic level. This suggests that most long-lived individuals are not only able to delay the onset of diseases but also that their capacity to completely avoid them is higher. Once again, other authors found that centenarians were able to completely escape diseases [12], and previous studies from our group demonstrated that centenarians from the Basque Country seemed to avoid or postpone aging-related diseases until the very end of their lives [24]. However, the morbidity profile of the rest of older adults in this region had not been characterized until now. The similarities in the trajectories of escapers shared by the youngest and oldest individuals of our cohort suggest that these two groups died with diseases that affected only one or few systems, instead of showing a complete-system decay. These results suggest that the “delayers” and “escapers” profiles of centenarians, closely linked to the “compression of morbidity” theory, could be expanded to other population groups. In fact, Franceschi et al. [13] proposed that centenarians were one of the extremes of the aging spectrum, and before them, there would be a continuum of different disease trajectories and consequent accelerated aging. In line with this, 81 years for men and 83 years for women were the lifespans at which individuals died with more systems affected, indicating that the life expectancy of this population involves a multisystemic condition [21] and that individuals that died outside this range had healthier profiles. Remarkably, at these ages, the prevalence of escapers for diseases of the circulatory system was at its minimum, along with diseases of the digestive and musculoskeletal systems. Therefore, a high percentage of these individuals seemed to die with cardiovascular disorders, which according to the World Health Organization constitute one of the leading causes of death [36]. When comparing these numbers to the SIDIAP study by Sol et al. [14], we saw that in their case, the minimum number of systems free of disease was located at 87 for men and 88 years for women, even though the life expectancy of the Catalan and Basque populations is very similar. This indicate that Basque individuals reached the minimum number of systems free of disease at an earlier lifespan. These differences could be associated with an earlier multisystemic decay in the Basque population. Consistent with this, when we compared the numbers of systems free of disease by decades, we observed that Basque individuals had more multimorbidity at all decades between 50 and 90 years. However, from 90 years onwards, they showed better health status. Furthermore, we observed that Basque individuals displayed earlier mortality, since the sample of individuals deceased between 50 and 69 years is higher (16.48% vs 12.33%) than in Catalonian population.

Concerning gender-specific patterns, we observed that women had fewer systems affected and showed a protective effect for most pathologies, especially for neoplasms, and diseases of the respiratory and genitourinary systems. Consistent with this, it has been shown that the incidence of most cancer types in men is far higher than in women [37]; however, there is some controversy about the prevalence of respiratory diseases, depending on the type of disease [38, 39]. On the other hand, women were more prone to nervous and musculoskeletal system diseases, which indeed have been reported to be more prevalent in women [40]. As for nervous system-related diseases, there is a lot of heterogeneity of prevalence among genders depending on the type of disease [41]. It is remarkable that among those who reached the highest lifespans, from 93 years onwards, men had fewer systems affected than women, suggesting that even though more women reached those ages, men did it in better conditions. Moreover, among centenarians, it has been reported that centenarian men are healthier, without severe diseases [42], and reach these ages with better physical function [35, 42]. These results could be linked to the male–female health survival paradox, with men showing a higher and earlier incidence of aging-associated diseases and consequent mortality, but also displaying healthier profiles if they survive at older ages. On the other hand, death rates for women are lower at all ages, but when compared to men at extreme ages, they had more diseases and disability [43], which was reflected in a higher prevalence of nervous and musculoskeletal system diseases. Remarkably, this pattern was not shown in the study by Sol et al., where long-lived women still exhibited more systems free of disease than men [14].

Overall, we found both similarities and differences in the outcomes of this study when compared to the one performed by Sol et al. using data from the SIDIAP [14]. Even though the patterns displayed by the different diseases in function of the lifespan, the trajectories of escapers, and the multisystemic evolution were similar, there were differences regarding gender- and disease-specific patterns. For instance, Basques seemed to be more prone to digestive and musculoskeletal diseases, and in the case of men, also to neoplasms and endocrine and metabolic diseases, when compared to the Catalan population. On the other hand, they had a smaller incidence of genitourinary and respiratory system diseases. These differences could be attributed to the different environments, health habits, and population customs. We have also found small differences in escapers trends; for example, in our cohort, long-lived men showed an increase in the proportion of escapers for circulatory system diseases that was not so pronounced in the SIDIAP study. Furthermore, the clustering of diseases according to the life free of disease was the analysis that showed the most notable differences, with fewer clusters and less smooth trajectories, which could also be attributed to our more limited sample size. In the case of the MFA, we demonstrated the ability of variables to explain lifespan, even though the contributions were different and we found two well-differentiated groups according to gender in our population. All these differences could also be due to the different periods studied, with the SIDIAP database exploring deceased individuals between 2006 and 2022 and our database with deceased individuals between 2014 and 2019.

This study has some limitations. Most of the diagnoses in our database were recorded through ICD-9 codes, and sometimes there was no exact match between ICD-9 and ICD-10 codes. Furthermore, there was a wide range of severity among the selected diseases, which could have distorted the analysis and the associations with lifespan. We do not have information related to the employment of the individuals involved on the study. Therefore, the healthy worker survivor effect [44] has not been characterized.

In summary, our results provide a detailed characterization of the trajectories of human aging in the Basque population and compare them to Catalonian population. It seems that Basque individuals showed an earlier multimorbidity which leads to mortality. Even though the reasons behind remain unknown, it is tempting to speculate that within the unique characteristics of Basque population, there might be some social-cultural and/or biological-genetic traits that facilitates the appearance of morbidity. In support of this idea, Basque individuals present higher prevalence of R1441G mutations in *LRRK2* gene, the most frequent cause of Parkinson's disease [17]. Additionally, our study reinforces the usefulness of individual-based approaches to unravel the heterogeneity of human aging process with different trajectories of lifespan and disease based on ethnicity. Moreover, our work together with the Sol et al. study highlight the benefit of the use of electronic health records in studying individual human aging trajectories.

## Supplementary Information

Below is the link to the electronic supplementary material.

Supplementary file1 (PPTX 1140 KB)

Supplementary file2 (DOCX 15 KB)

## Data Availability

The authors confirm that the data supporting the findings of this study are available within the article and its supplementary materials. The data that support the findings of this study are available from Basque Health Service but restrictions apply to the availability of these data, which were used under license for the current study, and so are not publicly available. Data are however available from the authors upon reasonable request and with permission of Basque Health Service.
