# Supplementary figures and images for: Analysis of sex-specific disease patterns associated with human lifespan
Source: GeroScience. 2025 Jan 15;47(3):2639–54. doi: 10.1007/s11357-024-01470-z (PMC12181580; doi:10.1007/s11357-024-01470-z)

## Slide 1
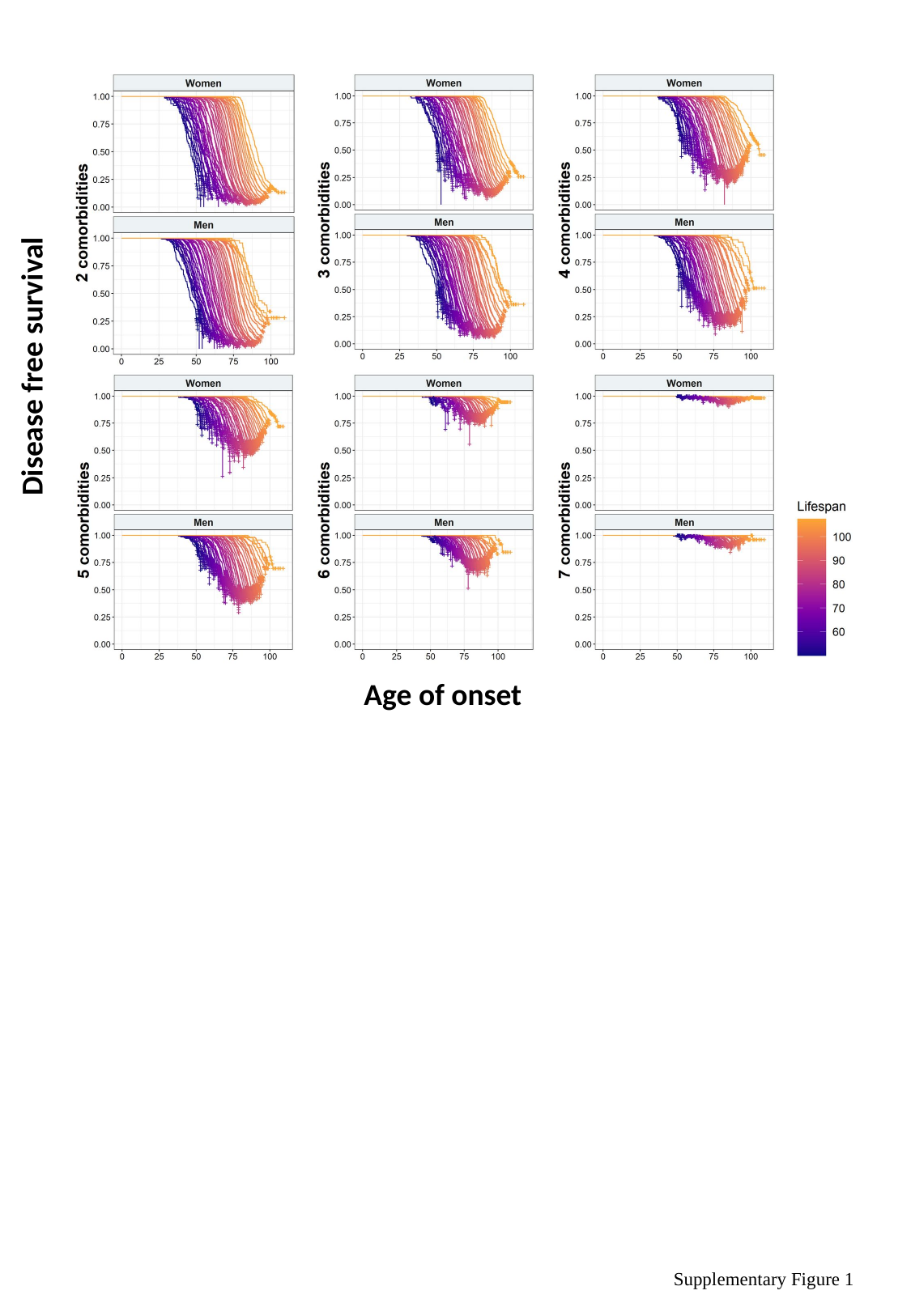

Disease free survival
Age of onset
Supplementary Figure 1

Supplement: Supplementary file 1 — Supplementary file1 (PPTX 1140 KB) [file 11357_2024_1470_MOESM1_ESM.pptx]
