# Supplementary material for: Analysis of sex-specific disease patterns associated with human lifespan
Source: GeroScience. 2025 Jan 15;47(3):2639–54. doi: 10.1007/s11357-024-01470-z (PMC12181580; doi:10.1007/s11357-024-01470-z)
Supplement: Supplementary file 2 — Supplementary file2 (DOCX 15 KB) [file 11357_2024_1470_MOESM2_ESM.docx]

**Supplementary Table 1.** Cox regression for each number of systems affected. Results are presented as HR with their respective 95% CI. Significant results are displayed in bold. * *p*<0.05, ** *p*<0.01, *** *p*<0.001.

| Number of systems affected | HR (95% CI) for age of death | HR (95% CI) for sex (women) | HR (95% CI) for age of death: sex (women) |
| --- | --- | --- | --- |
| 2 comorbidities | **0.871 (0.869-0.872)***** | **0.988 (0.968-1.009)** | **1.008 (1.006-1.010)***** |
| 3 comorbidities | **0.861 (0.859-0.863)***** | **0.965 (0.944-0.986)**** | **1.007 (1.004-1.009)***** |
| 4 comorbidities | **0.838 (0.836-0.840)***** | **0.911 (0.890-0.934)***** | 1.002 (0.999-1.004) |
| 5 comorbidities | **0.798 (0.795-0.801)***** | **0.832 (0.807-0.857)***** | 0.998 (0.995-1.002) |
| 6 comorbidities | **0.745 (0.740-0.751)***** | **0.709 (0.676-0.744)***** | 0.997 (0.991-1.002) |
| 7 comorbidities | **0.670 (0.657-0.683)***** | **0.633 (0.575-0.698)***** | 0.991 (0.979-1.003) |
| 8 comorbidities | **0.593 (0.551-0.638)***** | **0.472 (0.344-0.648)***** | 0.989 (0.948-1.032) |
